# Exercise-induced anaphylaxis with a cofactor of GIP/GLP-1 receptor agonist: A case report

**DOI:** 10.1016/j.jacig.2026.100751

**Published:** 2026-06-13

**Authors:** Albert C. Chong, Amani Elshaer, Maximiliano Diaz-Menindez, Jacqueline D. Squire, Alex M. Wonnaparhown, Catherine M. Freeman

**Affiliations:** a Department of Internal Medicine, Mayo Clinic, Scottsdale, Ariz; b Division of Pulmonary, Allergy, and Sleep Medicine, Mayo Clinic, Jacksonville, Fla; c Division of Allergy, Asthma, and Clinical Immunology, Mayo Clinic, Scottsdale, Ariz

**Keywords:** Tirzepatide, GLP-1 receptor agonist, GIP/GLP-1 receptor agonist, anaphylaxis, exercise-induced anaphylaxis, leukotriene

## Abstract

We report a patient who developed exercise-induced anaphylaxis after starting the dual glucose-dependent insulinotropic polypeptide/glucagon-like peptide-1 receptor agonist tirzepatide, who after discontinuing tirzepatide, experienced resolution of exercise-induced anaphylaxis.

Exercise-induced anaphylaxis (EIA) is an uncommon form of anaphylaxis characterized by symptoms provoked primarily by physical exertion.^1^ Symptoms occur during or shortly after exercise, improve with rest, and involve 2 or more organ systems. Although EIA most often occurs with vigorous activity, it may happen with mild exercise such as walking or yard work.^1^ In some cases, EIA is associated with cofactors that may precipitate anaphylaxis, such as medications, extreme temperatures, alcohol, and food allergens (in the case of food-dependent EIA).

In 2015, a European expert panel found that all proposed mechanisms for EIA lacked validity.^2^ Although a definitive mechanism remains poorly established, current hypotheses include increased gut permeability with translocation of mediators into the bloodstream; increased activity of tissue transglutaminase in the gut mucosa; redistribution of blood flow to different mast cell populations; release of cytokines (eg, IL-1β, IL-6, IL-8); and changes in plasma osmolality and acid-base balance, all of which may occur during exercise.^1^^,^^2^

Tirzepatide is a dual glucose-dependent insulinotropic polypeptide (GIP)/glucagon-like peptide-1 (GLP-1) receptor agonist that enhances insulin release and sensitivity alongside glucagon suppression. It is approved by the United States Food and Drug Administration for type 2 diabetes mellitus, chronic weight management, and obstructive sleep apnea in patients with obesity. A recent analysis of 39 million Americans eligible for GLP-1 receptor agonist treatment for obesity between 2021 and 2024 found that 2.3% of eligible patients were prescribed tirzepatide or semaglutide, with prescription prevalence tripling from 2023 to 2024.^3^ Although tirzepatide anaphylaxis has been reported,^4^ an association between EIA and GLP-1 receptor agonists has not been described previously.

We present the case of a 51-year-old man with obesity (body mass index 36 kg/m^2^), allergic rhinitis, asthma, and irritable bowel syndrome who was evaluated at our allergy and immunology clinic for recurrent exercise-related symptoms. After starting tirzepatide at a dose of 2.5 mg weekly for obesity, he experienced nausea and vomiting 24 hours after each injection for the first 5 weeks without exercise-related symptoms. During weeks 6 and 7, he experienced 4 episodes of facial flushing, lip swelling, palmar pruritus, chest pain, nausea, fecal urgency, and generalized weakness that occurred 5 to 15 minutes after 30 to 40 minutes of exercise.

The first 3 episodes followed high-intensity CrossFit exercise. The patient presented to the emergency department twice and improved rapidly with intravenous fluids, antiemetics, and famotidine. For the third episode, the patient was evaluated by his primary care provider, who started him on cetirizine and famotidine. The fourth episode occurred 5 hours before his visit to our clinic, following a moderate-intensity workout. In all cases, his symptoms started several minutes after exercise and resolved within 1 hour of stopping exercise. His symptoms occurred only in association with exercise, and he did not consume any food, alcohol, or nonsteroidal anti-inflammatory drugs several hours before exercise or between exercise and symptom onset. The patient had tolerated CrossFit for years without issue, had no prior anaphylaxis, and denied recent infections. Laboratory studies obtained 6 hours after symptom onset revealed an elevated urine leukotriene E_4_ level (Table I).

**Table I:** Laboratory values during tirzepatide treatment versus after discontinuation of tirzepatide treatment

Laboratory test	Value during tirzepatide treatment	Value after discontinuation of tirzepatide treatment	Reference range
Blood			
Leukocyte concentration	9.5 × 10^9^/L	7.0 × 10^9^/L	3.4-9.6 × 10^9^/L
Hemoglobin level	15.3 g/dL	16.2 g/dL	13.2-16.6 g/dL
Hematocrit level	46.3%	52.0%	38.3%-48.6%
Platelet count	266 × 10^9^/L	249 × 10^9^/L	135-317 × 10^9^/L
Eosinophil concentration	0.30 × 10^9^/L	Not obtained	0.03-0.48 × 10^9^/L
Serum			
Sodium level	143 mmol/L	140 mmol/L	135-145 mmol/L
Potassium level	4.2 mmol/L	4.2 mmol/L	3.6-5.3 mmol/L
Chloride level	106 mmol/L	103 mmol/L	98-108 mmol/L
Bicarbonate level	26 mmol/L	27 mmol/L	20-31 mmol/L
Blood urea nitrogen level	18 mg/dL	15 mg/dL	7-28 mg/dL
Creatinine level	1.06 mg/dL	1.03 mg/dL	0.68-1.37 mg/dL
Glucose level	110 mg/dL	83 mg/dL	70-99 mg/dL
Tryptase level	2.0 ng/mL	1.3 ng/mL	<11.5 ng/mL
Urine			
Leukotriene E_4_ level	427 pg/mg Cr	111 pg/mg Cr	≤104 pg/mg Cr
N-Methylhistamine level	97 μg/g Cr	90 μg/g Cr	30-200 μg/g Cr
2, 3-Dinor-11β-prostaglandin-F_2α_ level	520 pg/mg Cr	542 pg/mg Cr	<1802 pg/mg Cr

Initial serum tryptase and urine mast cell mediator levels were obtained 6 hours after onset of symptoms during tirzepatide treatment. Follow-up measurements were obtained 20 months after tirzepatide discontinuation.

*Cr*, Creatinine.

Given the temporal association with tirzepatide initiation, along with characteristic symptoms and laboratory evidence of mast cell degranulation, EIA with tirzepatide as a cofactor was diagnosed. The patient was counseled to discontinue tirzepatide, reduce exercise intensity, exercise with a partner, and carry an epinephrine autoinjector. Montelukast was added empirically alongside cetirizine and famotidine for an antileukotriene effect given an elevated urine leukotriene level.

Three weeks after discontinuing tirzepatide, the patient was able to perform moderate-intensity exercise with markedly diminished symptoms. At 3 months, he returned to high-intensity exercise without recurrence of anaphylaxis, and famotidine was discontinued. One year later montelukast was discontinued. At his 2-year follow-up, he continues to tolerate exercise without symptoms while taking cetirizine alone. Repeat laboratory tests at baseline showed a 4-fold drop in urine leukotriene level (Table I). The results of whole-extract and component-resolved testing for wheat IgE were negative (Table II).

**Table II:** Baseline whole-extract and component-resolved wheat IgE testing results

Laboratory test	Value	Reference range
Wheat IgE	0.36 kU/L	<0.70 kU/L
Gluten IgE	0.12 kU/L	<0.70 kU/L
ω5-Gliadin IgE	<0.10 kU/L	<0.10 kU/L

Here, we have reported the first case of EIA with a cofactor of GIP/GLP-1 receptor agonist. The patient’s reproducible symptoms involving multiple organ systems, occurring shortly after completion of exercise, and improving with rest are classic for EIA. Onset of these episodes following initiation of tirzepatide treatment and resolution with its discontinuation implicates tirzepatide as a cofactor to EIA. Only 64% of anaphylaxis cases feature elevated serum tryptase levels (>11.4 ng/mL) 1 to 2 hours after onset, with this proportion decreasing to 47% by hours 4 to 6.^5^ Meanwhile, urine leukotriene E4 levels peak at 3 to 6 hours after anaphylaxis onset, including for EIA.^6^ Elevated leukotriene E_4_ levels within 6 hours of symptom onset and normalization at follow-up after discontinuation of tirzepatide treatment provide strong biochemical evidence of tirzepatide-related EIA.

The gastrointestinal permeability hypothesis provides a plausible model for cofactors. Exercise alone is unlikely to meaningfully increase permeability at typical intensities; however, concurrent exposures that disrupt epithelial integrity (ie, aspirin or alcohol) may enhance antigen or mediator translocation and lower the threshold for anaphylaxis.^2^ Tirzepatide may function analogously by augmenting barrier changes and systemic exposure to these luminal factors. Exercise redistributes blood flow to skeletal muscle and skin, potentially delivering absorbed antigens to phenotypically distinct mast cell populations with lower activation thresholds.^2^ Separately, GLP-1 receptors are expressed on non–mast cell immune populations,^7^^,^^8^ suggesting that GIP/GLP-1 receptor agonism could also drive nonclassical EIA involving alternative immune cells.

A prior case of tirzepatide-associated anaphylaxis was reported by He et al,^4^ who described a biphasic anaphylactic reaction occurring approximately 20 minutes after first exposure to tirzepatide, without an exercise trigger. In that report, symptoms developed shortly after drug administration, which is consistent with tirzepatide acting as a primary trigger of an acute drug-induced anaphylactic reaction. In our patient, anaphylactic symptoms developed reproducibly only in association with exercise after multiple doses of tirzepatide and resolved following drug discontinuation. Together, these cases suggest different clinical contexts in which tirzepatide may contribute to anaphylaxis, either as a primary trigger or as a pharmacologic cofactor lowering the threshold for EIA.

EIA remains poorly understood. GIP/GLP-1 receptor agonists may represent a novel cofactor in EIA, although the underlying pathophysiologic mechanism requires further investigation. Discontinuing the agent appears to improve symptoms. Given a predicted 30 million users in the United States alone by 2030, and with the expanding indications and global use of GLP-1 receptor agonists, awareness of potential adverse reactions, including EIA, is essential.

Informed consent: Informed consent was obtained from the patient to publish this case report.

## Disclosure statement

Disclosure of potential conflict of interest: The authors declare that they have no relevant conflicts of interest.
